# Metabolite-driven dysfunction of intratumoral tertiary lymphoid structures: single-cell and spatial omics perspectives on cancer immune escape

**DOI:** 10.3389/fimmu.2026.1889313

**Published:** 2026-09-18

**Authors:** Yifeng Wang, Yan Li, Shujuan Wei, Shengguang Ding

**Affiliations:** 1 Thoracic Surgery Department, Nantong First People’s Hospital, Southeast University, Nantong, Jiangsu, China; 2 Thoracic Surgery Department, Beijing Shunyi District Hospital, Beijing, China; 3 Cardiovascular Surgery Department, Nantong First People’s Hospital, Southeast University, Nantong, Jiangsu, China; 4 Thoracic Surgery Department, Affiliated Nantong Clinical College of Nantong University, Nantong First People’s Hospital, School of Medicine, Nantong University, Nantong, Jiangsu, China

**Keywords:** adenosine, high endothelial venules, lactate, single-cell RNA sequencing, spatial transcriptomics, tertiary lymphoid structures, tryptophan metabolism, tumor metabolism

## Abstract

Intratumoral tertiary lymphoid structures (TLSs) are increasingly recognized as spatially organized immune niches associated with favorable prognosis and improved responses to immune checkpoint blockade. Yet TLS positivity is not synonymous with durable antitumor immunity. Many tumors contain lymphoid aggregates that are anatomically visible but developmentally arrested, poorly vascularized, depleted of germinal center-like activity, or uncoupled from effective plasma-cell and T-cell output. This discrepancy has shifted the field from a binary TLS-positive/TLS-negative view toward a state-based interpretation of TLS competence. Here, we propose that metabolite-driven remodeling of the tumor microenvironment is a major cause of intratumoral TLS failure. However, the current literature lacks a cell-type- and spatially resolved framework linking individual metabolites to disruption of defined TLS modules and distinguishing early developmental deviation from later functional collapse. Single-cell and spatial omics now show that productive TLSs depend on coordinated interactions among stromal organizer cells, HEV-like endothelium, mature dendritic cells, T follicular helper-like cells, B-cell lineages, plasma cells, and stem-like or progenitor exhausted T cells. These cellular modules are vulnerable to oxygen limitation, nutrient competition, lactate accumulation, extracellular adenosine, tryptophan catabolism, vascular stress, and suppressive B-cell states. A central goal of this review is to establish a hierarchy of mechanistic certainty across the metabolic drivers of TLS dysfunction. Tryptophan metabolism currently has the strongest direct TLS-specific spatial and perturbational evidence, whereas lactate, adenosine, hypoxia-associated vascular stress, lipid stress, and nucleotide metabolism are supported mainly by convergent immunometabolic and spatial evidence. On this basis, we outline biomarker-guided strategies to restore TLS competence, including inhibition of tumor-derived tryptophan metabolism to promote TLS maturation and enhance anti-PD-1 efficacy.

## Introduction

The conceptual frame of antitumor immunity is no longer confined to tumor-draining lymph nodes. Although draining lymph nodes remain indispensable for antigen transfer, cross-presentation, and the generation of systemic T-cell responses (1–3), many tumors also contain immune niches capable of sustaining local adaptive immunity (4, 5). Tertiary lymphoid structures (TLSs) are the most organized of these niches. They arise in chronically inflamed non-lymphoid tissues and may reproduce several principles of secondary lymphoid organs, including T-cell/B-cell compartmentalization, follicular dendritic cell (FDC) networks, plasma-cell differentiation, and high endothelial venule (HEV)-dependent lymphocyte entry (4–9). Their presence has repeatedly been linked with favorable prognosis and improved responsiveness to immune checkpoint blockade (10–13), which explains why TLSs have rapidly become one of the most clinically attractive tissue biomarkers of endogenous antitumor immunity.

This favorable association should not be overread. TLSs differ substantially in localization, cellular composition, vascular support, and maturation state (4, 5, 14, 15). A TLS embedded within the tumor bed and containing HEVs, FDCs, germinal-center-like B cells, Tfh-like help, and plasma-cell output is biologically different from a small lymphoid aggregate at the invasive margin (5–7, 16, 17). Presence-only scoring therefore collapses distinct biological states into a single category and may overlook differences in TLS density, spatial distribution, and maturation (4, 14, 15, 18). It also obscures an important clinical observation: some TLS-positive tumors remain resistant to checkpoint blockade, indicating that TLS presence is predictive but not determinative of therapeutic response (11–13). The more useful question is not whether a tumor contains TLSs, but whether those TLSs have become competent immune-organizing centers within an otherwise hostile tumor microenvironment.

Historically, the evaluation of tumor-associated TLSs relied mainly on routine histopathology, immunohistochemistry, and bulk transcriptomic immune signatures. These approaches were well suited to detecting lymphoid aggregates, estimating their abundance, and identifying broad maturation features, but they had limited ability to resolve cell-state heterogeneity, developmental trajectories, clonal relationships, or the spatial interactions among immune, stromal, vascular, and malignant-cell compartments. This methodological context naturally favored presence-, density-, and morphology-based classifications. The advent of single-cell and spatial omics has now made a state-based interpretation possible: single-cell approaches resolve the cellular states and lineage programs within TLSs, whereas spatial technologies reconnect these states to the tissue neighborhoods in which they develop and function. Together, these technologies reveal TLSs as dynamic multicellular ecosystems rather than static pathological landmarks (4, 5, 19). Studies in nasopharyngeal carcinoma (20), gastric cancer (21), head and neck squamous cell carcinoma (22), lung adenocarcinoma (23), renal cell carcinoma (7), and hepatocellular carcinoma (24) indicate that TLS heterogeneity is developmentally and ecologically encoded rather than merely morphological. In parallel, tumor immunometabolism has emerged as a major determinant of immune dysfunction. Lactate accumulation, extracellular adenosine, hypoxia, amino-acid catabolism, nucleotide turnover, and lipid remodeling are no longer viewed as passive by-products of tumor growth, but as active regulators of immune-cell fate, stromal behavior, vascular specialization, and therapeutic resistance (25–28). Their relevance to TLS biology is still being mapped, yet recent work suggests that metabolic stress can interfere with TLS initiation, maturation, maintenance, and output (24, 29, 30).

Here, we argue that metabolite-driven TLS dysfunction is an emerging mechanism of cancer immune escape. We deliberately distinguish direct TLS-specific causal evidence from mechanistic inference derived from broader tumor immunometabolism studies. Direct TLS-specific evidence is currently strongest for malignant-cell-driven tryptophan metabolism, which redirects immature TLSs toward a deviating trajectory and whose inhibition promotes TLS maturation and enhances the response to anti-PD-1 therapy. For lactate, adenosine, hypoxia-associated vascular dysfunction, and lipid remodeling, primary experimental studies support effects on individual TLS-relevant immune or vascular modules, but direct TLS-focused perturbation evidence remains limited; these studies are discussed in the corresponding pathway-specific sections below (25, 26, 28, 29, 31–34). This distinction is important for translational development: a useful TLS biomarker must report not only structure and abundance, but also location, cellular state, vascular competence, developmental trajectory, and metabolic permissiveness. Importantly, productive TLSs should not be regarded as metabolically neutral structures. Their development and function require a metabolically permissive niche in which oxygen and nutrient delivery, metabolite clearance, and cell-intrinsic mitochondrial and redox fitness remain sufficient to support coordinated endothelial, stromal, dendritic-cell, T-cell, and B-cell functions. Metabolic permissiveness therefore denotes active metabolic support rather than the absence of metabolic regulation. By contrast, excessive accumulation of suppressive metabolites or failure of cellular metabolic adaptation can redirect a visible TLS toward a stalled or dysfunctional state. **Figure 1** summarizes how the balance between metabolic support and metabolic pressure may direct visible intratumoral TLSs toward productive or stalled immune states.

**Figure 1:**
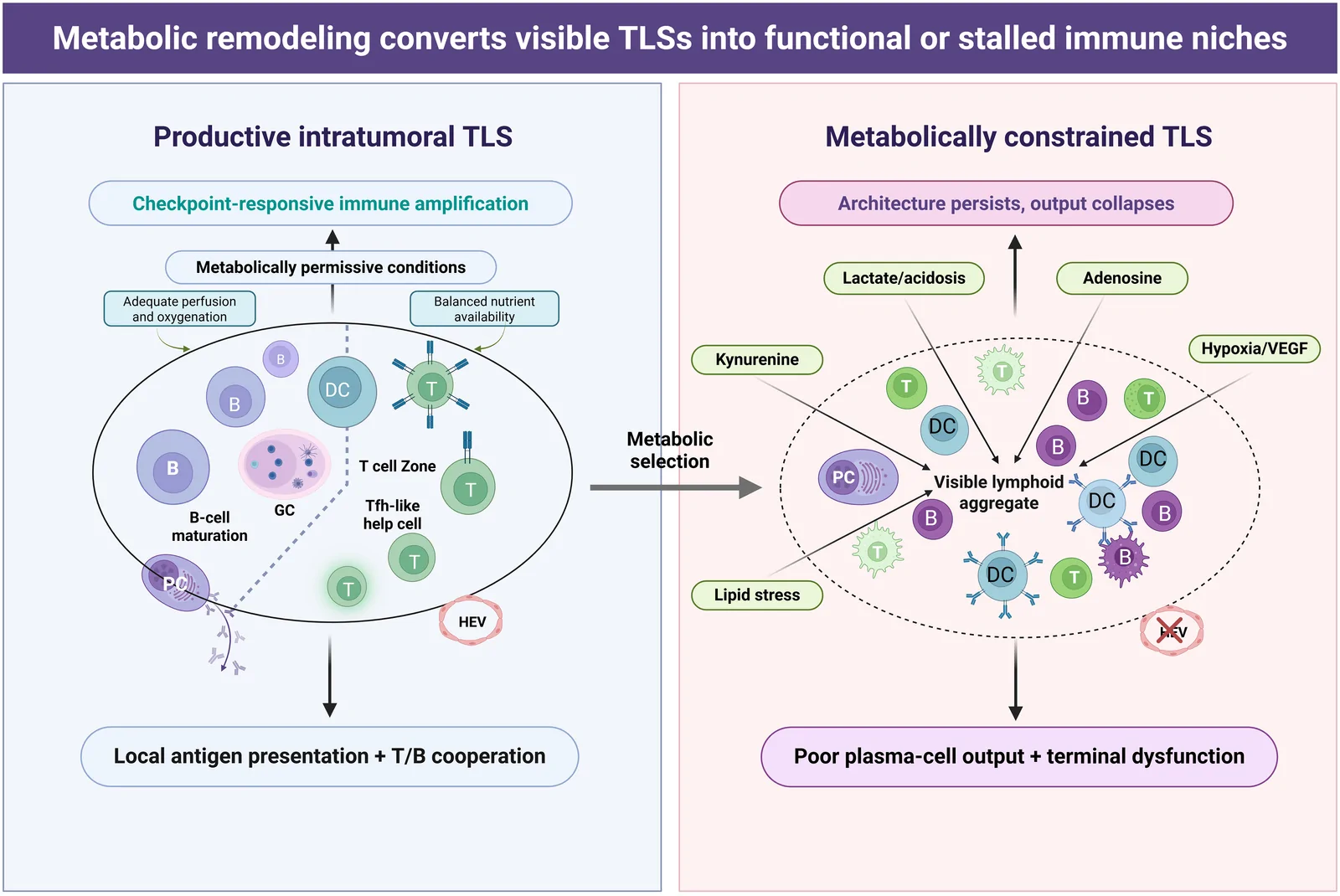
Conceptual model of metabolite-driven intratumoral TLS dysfunction. Productive intratumoral TLSs integrate HEV-mediated lymphocyte entry, dendritic-cell priming, Tfh-B-cell cooperation, germinal-center-like B-cell maturation, plasma-cell output, and checkpoint-responsive T-cell states. Metabolically constrained TLSs may remain histologically visible but lose vascular competence, antigen-presenting coordination, humoral output, and effective T-cell function. Productive TLS function is supported by metabolically permissive conditions, including adequate perfusion and oxygenation and balanced nutrient availability.

## TLSs as local immune organs in cancer

TLSs arise in chronically inflamed non-lymphoid tissues through coordinated interactions among inflammatory cytokines, lymphoid tissue inducer-like signals, stromal organizer cells, homeostatic chemokines, and specialized vasculature (35–38). Canonical chemokine axes, particularly CXCL13-CXCR5 and CCL19/CCL21-CCR7, together with lymphotoxin- and TNF-family signaling, sustain immune-stromal crosstalk and drive progressive structural organization (35–39). Although TLSs are not encapsulated like lymph nodes, mature TLSs reproduce the key organizational principles of secondary lymphoid organs, including compartmentalized T-cell and B-cell zones, FDC networks, antigen-presenting cells, and HEVs that support continuous lymphocyte recruitment (4–6, 37, 38, 40). TLSs should therefore be viewed not as passive inflammatory aggregates, but as adaptive tissue structures whose architecture and function are continuously shaped by the tumor microenvironment (41, 42).

This organization is functionally important because it enables local antitumor immunity. In the classical cancer-immunity cycle, priming of tumor-reactive lymphocytes is generally assigned to draining lymph nodes (43, 44). In TLS-bearing tumors, however, several of these steps can be reproduced *in situ*. Mature dendritic cells can capture and present tumor antigens locally, T cells can be activated within organized T-cell zones, and B cells can accumulate in follicle-like regions where they receive T-cell help, undergo class switching and affinity maturation, and differentiate into plasmablasts or plasma cells (7, 45–47). This supports the concept of an intratumoral immunity cycle, in which antigen release, presentation, T-cell activation, B-cell maturation, antibody production, and renewed antigen presentation reinforce one another within the tumor microenvironment (5, 7, 47, 48). HEVs are central to this circuit because they provide specialized entry sites for circulating lymphocytes and thereby convert the tumor bed into an immune-accessible niche (46, 49–51). At the same time, mature dendritic cells and germinal center-like B-cell regions allow TLSs to coordinate cellular and humoral immunity in a self-reinforcing local network (5, 7, 45, 47, 48).

Single-cell and spatial studies have strengthened this view by showing that TLSs are multicellular immune ecosystems rather than simple sites of lymphocyte accumulation. In several tumor types, including nasopharyngeal carcinoma, endometrial cancer, melanoma, renal cell carcinoma, and head and neck cancer, TLSs are associated with CXCL13^+^ cancer-associated fibroblasts, stem-like CXCL13^+^CD8^+^ T cells, B cells, and T follicular helper (Tfh) cells, suggesting that they act as local developmental niches that actively instruct antitumor immunity (7, 11, 20, 22, 52, 53). Importantly, however, not all TLSs are biologically equivalent. Their antitumor relevance depends far more on functional maturity than on histological visibility alone (4, 5, 18, 19, 41, 42, 48, 54, 55). Poorly organized lymphoid aggregates may contain infiltrating lymphocytes but fail to support productive immunity if they lack HEVs, FDC networks, clear T/B-cell segregation, or germinal center-like activity (41, 42, 54). Conversely, even relatively small intratumoral TLSs may exert strong biological effects when they contain activated B cells, plasma cells, Tfh-like helper cells, competent dendritic cells, and vascular support. This distinction provides the conceptual basis for viewing TLS dysfunction not simply as reduced immune infiltration, but as a form of local immune-organ failure within the tumor microenvironment. Although mature intratumoral TLSs are usually associated with productive antitumor immunity, lymphoid organization should not be assumed to be uniformly protective across all tumor contexts, as ectopic lymphoid structures can acquire different biological roles depending on tissue setting and niche composition (56).

## From TLS presence to TLS state

Recent pathology-oriented work in NSCLC reinforces the transition from presence-based to multidimensional TLS assessment. In particular, Berthe et al. developed a prognostic TLS score integrating TLS relative area, B-cell density, and the density of CD21-positive/CD23-negative follicular dendritic cells (54, 57, 58). For many years, TLS research in cancer was dominated by pathology-based scoring systems that classified TLSs according to their presence, density, and degree of maturation (4, 16, 18, 54, 58). This emphasis partly reflected the resolution of the available methods, which could identify lymphoid structures and broad immune signatures but could not reliably reconstruct their cell-state composition, developmental trajectories, and spatial ecosystem context. These schemes established TLSs as clinically relevant biomarkers, but they are no longer sufficient to explain the heterogeneity revealed by modern profiling technologies. A simple TLS-positive versus TLS-negative classification cannot capture lineage transitions, chemokine circuitry, clonal dynamics, metabolic constraints, or the spatial relationship between TLSs and malignant-cell programs (5, 19, 20, 24, 59). A more informative framework is to consider TLSs in terms of state rather than mere presence. In this view, TLSs lie along a continuum defined by structural organization, vascular competence, spatial localization, cellular composition, and microenvironmental permissiveness (48, 54, 59–61) (**Figure 2**). Accordingly, a sparse lymphoid aggregate, a follicle-like structure lacking a germinal center, and a fully organized intratumoral niche containing HEVs, FDCs, germinal center B cells, plasma-cell output, and coordinated T-cell help should not be treated as functionally equivalent (48, 54, 55). The critical question is therefore not simply whether a TLS exists, but which developmental and functional state it occupies.

**Figure 2:**
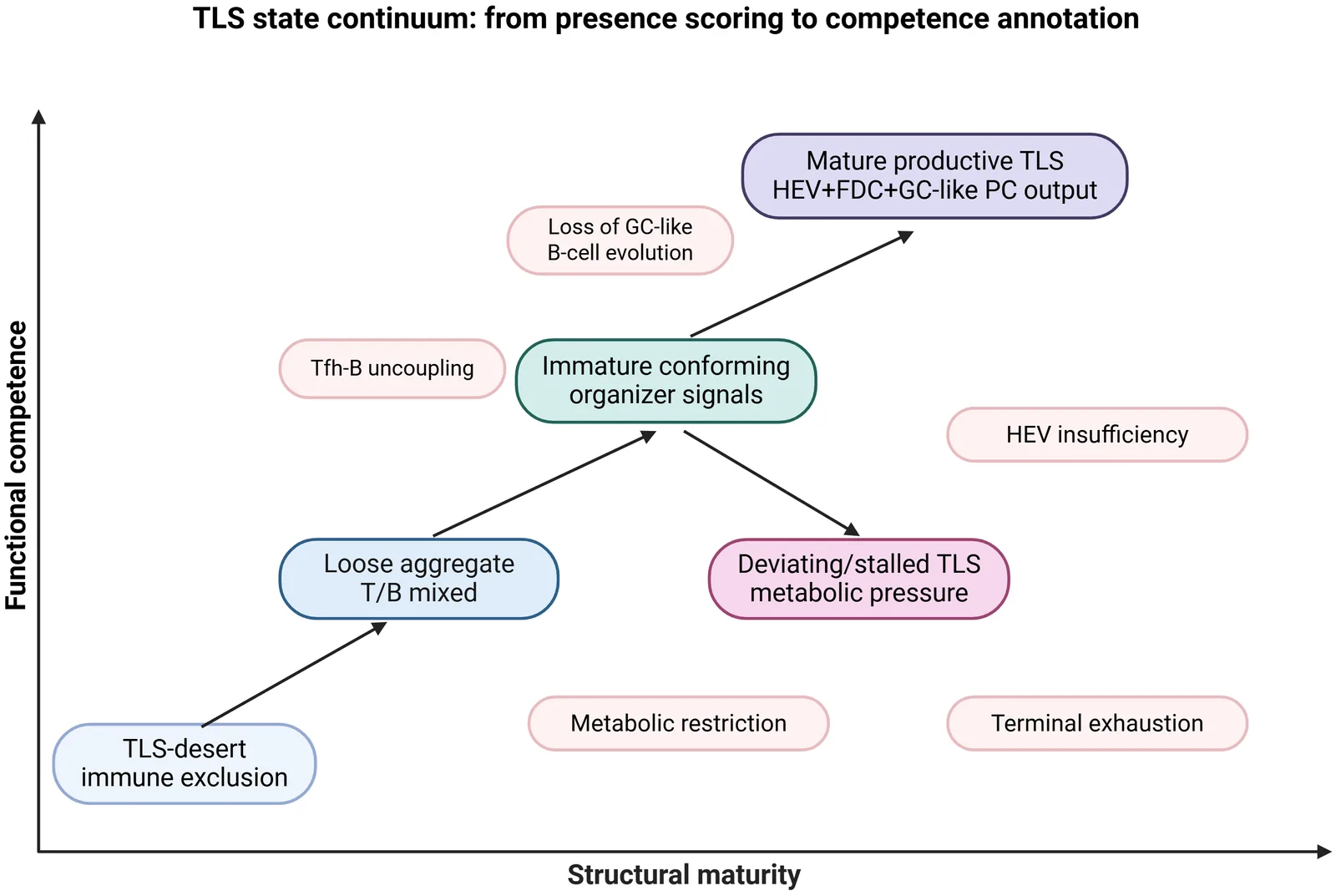
State-based interpretation of TLSs. TLSs should be placed on axes of structural maturity and functional competence rather than categorized simply as present or absent. Metabolic pressure can divert immature structures toward deviating or stalled states despite persistent lymphoid morphology.

Evidence from pathology and early immunotherapy studies had already pointed toward this conceptual shift. In lung squamous cell carcinoma, prognostic value was determined more by germinal center formation than by TLS number alone (54). In esophageal squamous cell carcinoma, mature TLSs were associated with stronger helper T-cell signatures, plasma-cell programs, memory B-cell features, and greater intratumoral CD8^+^ T-cell infiltration (62). Early immunotherapy studies reached a similar conclusion. In melanoma, Cabrita et al. demonstrated that TLS-rich tumors were associated with improved immunotherapy outcomes, whereas T cells in tumors lacking TLSs displayed a more dysfunctional molecular phenotype (11). Across melanoma, renal cell carcinoma, and sarcoma, the association of B cells and TLSs with favorable immunotherapy outcomes further supported the idea that TLSs are active immune niches rather than incidental inflammatory bystanders (12). More recently, single-cell and spatial profiling have made the distinction between TLS presence and TLS state much clearer. In renal cell carcinoma, intratumoral TLSs support a complete trajectory of B-cell maturation toward plasma cells and local antibody production associated with immunotherapy benefit (7). In nasopharyngeal carcinoma, gastric cancer, and head and neck squamous cell carcinoma, productive TLS ecosystems are characterized by coordinated stromal organizers, HEV-associated endothelial programs, Tfh-like help, active B-cell differentiation, and dendritic-cell support (20, 21, 63). By contrast, non-productive TLSs may be developmentally arrested, spatially excluded, metabolically constrained, or compositionally distorted (24, 59, 60, 63, 64).

This state-based framework is especially compelling in hepatocellular carcinoma, where spatial transcriptomics resolved immature intratumoral TLSs into at least two trajectories: a conforming state that retains niche functions resembling mature TLSs and a deviating state that does not (24). Importantly, this divergence was linked to a tryptophan-enriched metabolic microenvironment imposed by malignant cells (24), suggesting that TLS failure can be metabolically encoded before overt structural collapse becomes visible. In other words, a tumor may remain TLS-positive while already shifting into a dysfunctional state. Once TLS state is taken into account, several previously puzzling observations become easier to interpret. Presence-only scoring cannot determine whether HEVs are mature, whether germinal centers are active, whether B cells are undergoing class switching and plasma-cell differentiation, whether T-cell help remains stem-like rather than terminally dysfunctional, or whether the surrounding niche is metabolically permissive (24, 48, 54, 62–66). It also fails to capture upstream constraints that may erode TLS competence before visible TLS loss occurs. For example, sensory nerves can impair HEV maturation and mature TLS formation in melanoma (67), and metabolites such as lactate, adenosine, and kynurenine are likely to act in similarly upstream ways. Together, these findings argue for a multidimensional framework that incorporates TLS location, vascular and stromal support, immune-cell states, evidence of productive plasma-cell differentiation, and the metabolic conditions of the surrounding niche. These features are not merely descriptive; they determine whether a TLS acts as an active site of local antitumor immunity or persists as a stalled lymphoid remnant within a hostile microenvironment.

As the evidence above suggests, the field now requires a comparative framework that explains why morphologically recognizable TLSs can be prognostically favorable in some settings yet developmentally stalled or therapeutically uninformative in others. Across tumor types, the most informative variables repeatedly extend beyond simple TLS counts to include germinal center activity, HEV competence, stromal organization, helper T-cell support, plasma-cell output, and the broader spatial or metabolic niche that conditions these programs. **Table 1** synthesizes these cross-tumor observations and highlights a common principle: TLS significance is best interpreted as a state-dependent property shaped by developmental maturity and ecosystem context rather than by presence alone. Viewed together, these studies converge on a single interpretive shift: TLSs should be analyzed as ecological states rather than binary histological features. This cross-tumor synthesis provides the rationale for the next section, in which single-cell and spatial omics are considered as the key tools for resolving the stromal, vascular, myeloid, T-cell, and B-cell programs that distinguish productive from non-productive TLS ecosystems.

**Table 1:** Cross-tumor evidence supporting state-based interpretation of tertiary lymphoid structures.

Tumor context or study type	TLS-relevant observation	Interpretation for TLS state	Relevant translational message
NSCLC/lung squamous cell carcinoma	Germinal-center formation and mature organization improve the prognostic value of TLS assessment (refs. 54, 57, 58).	Structural maturity is more informative than TLS number alone.	Report TLS area, B-cell density, FDC organization, HEV status, and GC-like features instead of binary TLS positivity.
Melanoma and early ICI cohorts	TLS-rich tumors associate with improved checkpoint blockade outcomes, whereas TLS-poor tumors show more dysfunctional T-cell states (refs. 11, 12).	TLSs act as local immune niches but require a compatible T-cell state.	Use TLS maturity together with T-cell exhaustion/progenitor markers for ICI stratification.
Renal cell carcinoma	Intratumoral TLSs support B-cell maturation toward plasma cells and local antibody-producing programs (ref. 7).	Productive TLSs can sustain humoral antitumor immunity.	Include plasma-cell output and antibody-related programs in TLS scoring.
Head and neck squamous cell carcinoma	Tfh-like progenitor exhausted CD4^+^ T cells support B-cell maturation and CD8+ T-cell activation (ref. 63).	Helper T-cell state is a central module of TLS competence.	Annotate Tfh-like/progenitor exhausted CD4^+^ cells rather than reporting total CD4^+^ infiltration only.
Nasopharyngeal and gastric cancers	Single-cell and spatial profiling identifies coordinated stromal organizers, HEV-associated endothelial states, Tfh-like cells, B cells, plasma cells, and dendritic cells (refs. 20, 21).	TLS function is spatially and ecologically encoded.	Pair single-cell references with spatial tissue maps to distinguish intratumoral, peritumoral, and TLS-desert states.
Hepatocellular carcinoma	Spatial transcriptomics resolves conforming versus deviating immature TLS trajectories linked to tryptophan-rich malignant-cell niches (ref. 24).	Metabolism can redirect TLS maturation before morphology disappears.	Measure tryptophan/kynurenine programs and test metabolic redirection with anti-PD-1 therapy.
NSCLC with mature TLSs	Spatially resolved studies show that even mature TLS-positive tumors may resist immunotherapy when embedded in suppressive ecosystems (ref. , 64).	Mature morphology is insufficient without a permissive surrounding niche.	Combine TLS annotation with stromal, myeloid, metabolic, and T-cell dysfunction maps.
Bladder cancer/suppressive B-cell states	IGLL5^+^ B cells can disrupt HEV-associated lymphotoxin signaling, TLS integrity, and immunotherapy efficacy (ref. , 72).	B-lineage cells are not uniformly protective.	Distinguish productive germinal-center/plasma-cell programs from suppressive B-lineage states.

## Single-cell and spatial omics redefine TLS heterogeneity

Single-cell methods identify the immune, stromal, vascular, and malignant-cell states that build or destabilize TLSs and can also infer cell-intrinsic metabolic programs from the expression of metabolic enzymes, transporters, receptors, and mitochondrial or redox-response genes. Spatial platforms then place these cellular and inferred metabolic states back into their tissue coordinates and reveal whether they are coordinated, excluded, fragmented, or exposed to metabolically permissive or constrained neighborhoods. Cellular metabolism is therefore an integral, rather than intentionally excluded, component of the proposed framework. However, transcriptomic signatures indicate metabolic potential and should not be interpreted as direct measurements of metabolite abundance or pathway flux. **Figure 3** summarizes this integrated cellular, spatial, clonal, and metabolic workflow for TLS state annotation.

**Figure 3:**
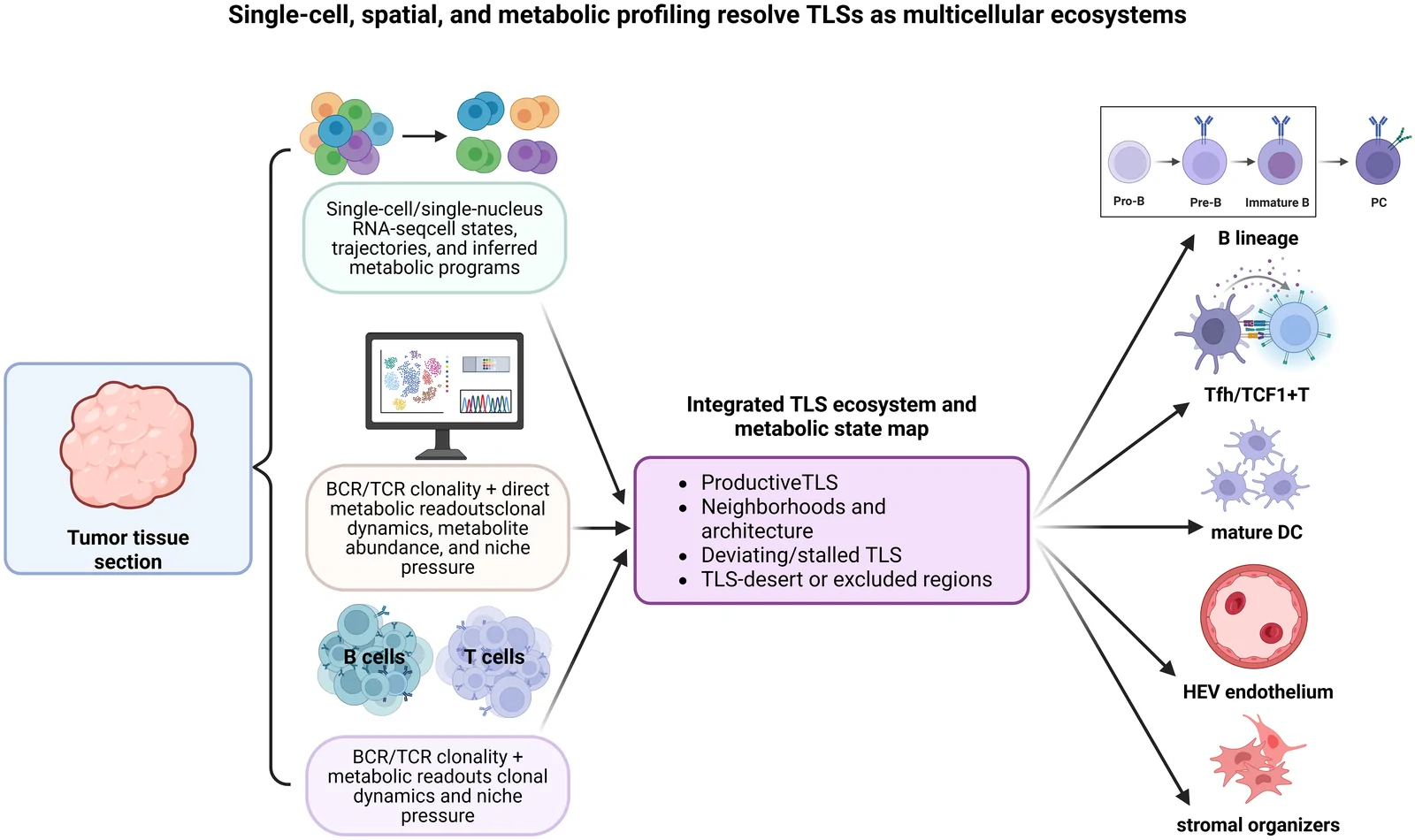
Integrated single-cell, spatial, clonal, and metabolic workflow for resolving TLS ecosystem states. Single-cell or single-nucleus sequencing defines immune, stromal, vascular, and malignant-cell states and permits inference of cell-type-specific metabolic programs based on the expression of metabolic enzymes, transporters, receptors, and mitochondrial or redox-response genes. Spatial transcriptomics and multiplex imaging reconnect these cellular and inferred metabolic states to tissue architecture and metabolically permissive or constrained neighborhoods. Integration with BCR/TCR repertoire profiling and extracellular metabolite-sensitive readouts helps distinguish productive, conforming, deviating or stalled, and TLS-desert ecosystem states. Transcriptomic metabolic signatures reflect metabolic potential and should not be interpreted as direct measurements of metabolite abundance or pathway flux.

### Single-cell dissection of immune and stromal modules

Across TLS-containing tumors, single-cell studies identify a continuum of B-cell states, including naive-like, memory-like, germinal center-like, activated, plasmablast, and terminal plasma-cell populations (68–70). This spectrum supports the view that TLSs can act as local sites of humoral evolution. However, newer data also caution against treating all B-lineage cells as beneficial (71, 72). B cells and plasma cells can support antigen presentation, antibody production, and T-cell cooperation (7, 12, 48, 73), but selected B-cell states may also weaken TLS integrity or contribute to immune suppression (71, 72). T-cell profiling adds another layer of heterogeneity, including stem-like or progenitor-like TCF1+ states, progenitor exhausted and terminally exhausted populations, Tfh-like cells, Treg cells, and tissue-resident memory subsets (20, 63, 74–77). Myeloid cells, particularly mature migratory dendritic cells, sustain antigen presentation and T-cell priming (45, 46), while specialized fibroblasts and perivascular organizer-like cells expressing CCL19, CCL21, and CXCL13 promote lymphocyte recruitment, compartmental organization, and TLS maturation (20, 78, 79). Productive TLS function therefore emerges from coordinated interactions among B-lineage cells, helper and exhausted T-cell states, antigen-presenting myeloid cells, stromal organizers, and vascular gateways.

Integrative studies reinforce this systems-level view by showing that TLS heterogeneity is determined less by abundance alone than by the quality of the surrounding circuitry. Pan-cancer atlases demonstrate that intratumoral B-cell responses follow hierarchical lineage programs across tumor types, including naive-to-germinal center transitions, plasmablast and plasma-cell differentiation, clonal expansion, and chromatin remodeling, further supporting the concept that TLSs are biologically active sites of local humoral evolution (68–70). At the same time, single-cell and spatial studies show that productive TLS function depends on a supportive immune and stromal context. In hepatocellular carcinoma, immature TLSs diverge into conforming and deviating trajectories under malignant cell-driven tryptophan pressure (24). In nasopharyngeal carcinoma, coordinated TLS ecosystems comprise CXCL13^+^ fibroblasts, stem-like CXCL13^+^CD8^+^ T cells, Tfh cells, B cells, and plasma cells, highlighting the need for cooperation among stromal organizers, helper T cells, and differentiating B-lineage populations (20). In renal cell carcinoma, TLS-positive tumors show B-cell maturation toward plasma cells and evidence of local humoral antitumor immunity (7), whereas in breast cancer, fibroblast subsets and macrophage programs appear to influence lymphocyte recruitment and niche quality (80). Similarly, in head and neck squamous cell carcinoma, progenitor exhausted CD4^+^ T cells with Tfh-like properties support both B-cell maturation and CD8^+^ T-cell activation (63). Notably, response to PD-1 blockade in renal cell carcinoma depends not simply on TLS presence, but on high TLS abundance together with a lower burden of terminally exhausted tissue-resident CD8^+^ T cells (81–84). Thus, even TLS-rich tumors may remain clinically refractory when the surrounding T-cell compartment is locked in a deeply dysfunctional state.

### Spatial reconstruction of TLS ecosystems

Although dissociative single-cell approaches have greatly advanced TLS research, they inevitably disrupt tissue architecture and therefore cannot fully capture the spatial logic that underlies TLS function. In TLS biology, spatial organization is not just descriptive but causal, because TLSs are embedded within and influenced by tumor nests, vascular territories, fibroblastic tracks, necrotic regions, and metabolically stressed niches (85–87). These relationships shape whether local immune programs are sustained, amplified, or diverted into dysfunctional states. Spatial transcriptomics and multiplex imaging provide this missing layer of information. In nasopharyngeal carcinoma, integrated mapping of TLSs and adjacent tumor aggregates identified coordinated immune and stromal ecosystems associated with both disease progression and response to immunotherapy (20). Likewise, in gastric cancer, the prognostic value of TLSs depended more on spatial localization and ecological context than on density alone (21). Intratumoral TLS-rich regions were enriched in CXCL13^+^ T cells, CXCR5^+^ germinal center B cells, activated dendritic cells, and HEV-associated endothelial programs, whereas peritumoral TLS states and TLS-desert regions reflected distinct immune environments (21). These observations indicate that TLS function cannot be understood simply by counting lymphoid aggregates; it also requires analysis of where TLSs are positioned and how they interact with surrounding tumor and stromal elements. Spatial context may also explain why even morphologically mature TLSs do not always translate into therapeutic responsiveness, as recent work in NSCLC showed that mature TLS-positive tumors can remain resistant when embedded in suppressive spatial ecosystems (64).

Spatial approaches also make it possible to reconstruct TLS developmental continua directly *in situ*, revealing how architecture, cellular composition, and metabolic context converge to shape TLS fate. In hepatocellular carcinoma, spatial analyses identified immature, conforming, deviating, and mature TLS-like structures and showed that metabolic context contributes to deviation from canonical maturation trajectories (24, 88). Importantly, spatial studies also indicate that TLS activity extends beyond the lymphoid aggregate itself. In renal cell carcinoma, plasma cells generated within TLSs appear to disseminate along fibroblastic tracks into adjacent tumor beds, where IgG-coated tumor cells and malignant-cell apoptosis support a model of local antibody-mediated antitumor immunity (7). In diffuse glioma, some TLS-like structures lacking classical germinal center morphology nonetheless exhibited clonal T- and B-cell expansion, plasma-cell generation, and dendritic-cell/T-cell interactions associated with improved survival (89). Collectively, these findings show that TLS heterogeneity operates across several interconnected dimensions, including localization, architecture, cellular composition, developmental state, and metabolic conditioning. Immune escape may therefore reflect not only the absence of TLSs, but also the persistence of morphologically recognizable TLS ecosystems that are spatially misplaced or functionally miswired. Methodologically, this interpretation will benefit from explicit integration of single-cell references, spatial transcriptomic maps, and histological morphology rather than relying on one modality alone (6).

### TLSs as ecological, not merely structural, entities

TLSs should not be viewed as static histological ornaments. Their biological significance depends on whether they function as living immune ecosystems capable of sustaining antigen capture, lymphocyte entry, germinal center-like B-cell evolution, T-cell help, plasma-cell differentiation, and communication with the surrounding tumor bed (5, 41, 42, 90). Recent spatial and single-cell studies sharpen this distinction by showing that TLS competence is determined not only by lymphocyte abundance, but also by stromal organizers, vascular gateways, immune-cell state composition, and metabolic context (20, 24, 69, 89). From this perspective, a TLS is best understood as an ecological unit embedded within either a permissive or a hostile niche. Stromal and vascular elements are therefore not passive scaffolds, but active regulators of TLS emergence and maturation. For example, CCL19-producing fibroblasts in colorectal cancer liver metastasis promote lymphocyte trafficking, TLS neogenesis, and plasma-cell responses (79), whereas pan-cancer spatial atlases have identified CCL19^+^ perivascular organizer-like cells associated with TLS formation and HEV-like endothelial states (91). In pancreatic cancer, IL-33-activated ILC2s can also promote TLS formation through lymphotoxin-related programs (92). Successful TLS assembly thus depends on the quality of stromal organizers, vascular conduits, and incoming cellular fluxes. Importantly, however, ecological framing also implies that TLSs are not uniformly beneficial simply because they are organized. In most solid tumors, mature intratumoral TLSs are associated with favorable immune activity, yet earlier work in hepatocellular carcinoma showed that ectopic lymphoid structures can also function as microniches for tumor progenitor cells (56). Likewise, more recent spatial work indicates that even mature TLS-positive tumors may remain clinically resistant when the surrounding ecosystem is dominated by suppressive stromal and dysfunctional immune programs (64). Lymphoid organization alone is therefore insufficient to define biological function.

The same ecological logic applies to lymphocyte state. Mature TLSs are not simply larger lymphoid aggregates, but spatially organized immune niches in which B-cell follicles, T-cell zones, follicular dendritic-cell networks, HEV-associated lymphocyte trafficking, and germinal-center reactions can support local antigen presentation, affinity maturation, and adaptive immune amplification (4, 5, 48, 54). Tumors lacking mature TLSs may still contain sparse B cells or immunoregulatory B-cell populations; therefore, B-cell abundance alone should not be equated with productive TLS biology (69, 93). Consistently, pan-cancer single-cell analyses indicate that tumor-infiltrating B cells can follow divergent germinal-center-like versus extrafollicular trajectories, with extrafollicular programs linked to worse clinical outcomes and resistance to immunotherapy (69). Immature TLSs should therefore not be interpreted as merely smaller versions of mature TLSs, but as potentially divergent ecological states with distinct developmental trajectories and functional outputs (5, 54, 55). This framework also explains why TLS meaning is context-dependent at the whole-tumor level. A tumor may be TLS-positive yet still fail to be TLS-effective if HEV-like endothelium is absent, organizer stromal-cell programs are unstable, dendritic-cell support is inadequate, or metabolic constraints within the tumor microenvironment prevent the maturation, maintenance, and systemic propagation of adaptive immunity (4, 5, 29, 41, 45, 49).

The clearest direct evidence for such ecological derailment comes from hepatocellular carcinoma, where a tryptophan-enriched microenvironment drives immature TLSs away from productive maturation (24). Broader immunometabolic studies support the same principle. Tumor-derived lactate can induce tolerogenic dendritic-cell programs (94), available evidence suggests that hypoxia-adenosine signaling may restrict lymphocyte trafficking and alter humoral programs (95, 96), and nutrient competition can impair B-cell fitness within the tumor microenvironment (97). Importantly, these mechanisms may not abolish TLS formation altogether. Instead, they may corrupt the surrounding niche so that lymphoid organization emerges but fails to complete productive maturation. Under this model, TLS failure is often not a failure of assembly, but a failure of ecological maintenance and developmental completion.

## HEVs as vascular gatekeepers of functional TLSs

Among the structural features of TLSs, HEVs deserve particular attention. These specialized post-capillary venules are essential for lymphocyte trafficking in secondary lymphoid organs and are also a defining vascular component of productive intratumoral TLSs (49, 51, 98). Their presence indicates that a tumor has established a vascular program compatible with organized local immunity, as tumor-associated HEVs correlate with T- and B-cell infiltration, immune-enriched tumor regions, and favorable clinical outcome in several solid tumors (6, 49, 99). HEVs matter for at least three reasons. First, they sustain cellular influx and replenishment; a TLS that cannot recruit fresh lymphocytes is unlikely to maintain growth, maturation, or durable function (49–51, 98). Second, HEVs reflect a specialized endothelial state distinct from generic tumor angiogenesis, characterized by immune-recruiting post-capillary venule programs rather than merely abnormal pro-angiogenic tumor vasculature (49–51, 76). Third, they connect intratumoral immune niches to systemic immunity and may therefore help explain why HEV-rich tumors often respond better to immunotherapy (49, 50, 76). Primary studies have shown that tumor-associated HEVs are major sites of lymphocyte arrest, extravasation, and entry and that increasing HEV frequency and maturation enhances the infiltration of stem-like CD8-positive T cells and responsiveness to checkpoint blockade (50). Immunotherapy-induced endothelial reprogramming can also generate HEV-like vessels that support TCF1-positive T-cell niches (76), while spatial studies have identified coordinated organizer-cell–endothelial programs associated with HEV-like states (91). These findings establish the functional importance and plasticity of tumor-associated HEVs. They also raise the possibility that a hostile metabolic environment may interfere with HEV specialization or maintenance. However, direct evidence that hypoxia, lactate, extracellular adenosine, or VEGF-driven stress specifically disrupts HEV differentiation within TLSs remains limited. We therefore regard HEV insufficiency as a plausible metabolic bottleneck in TLS maturation rather than an established causal consequence of any single metabolite (**Figure 4**).

**Figure 4:**
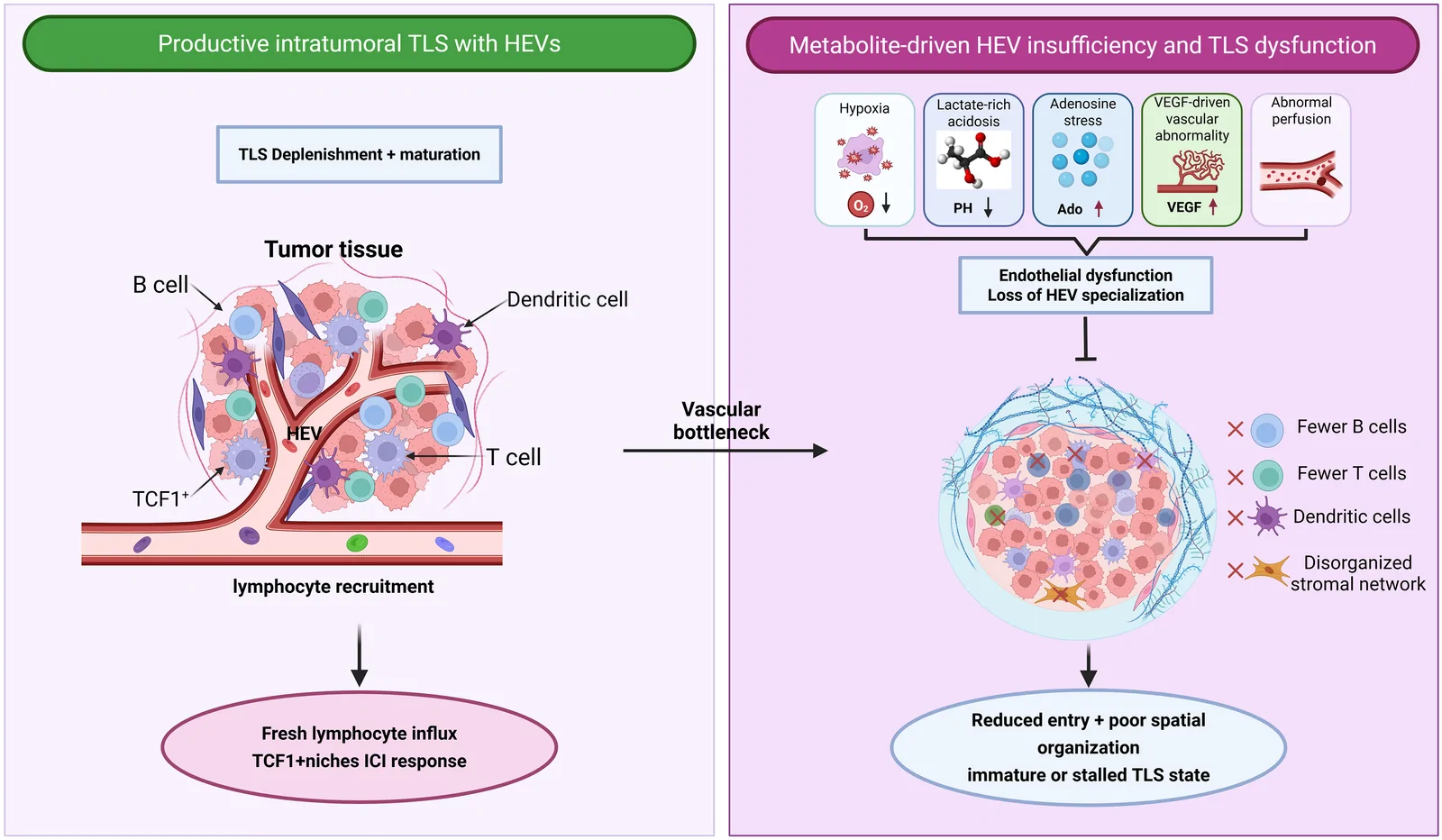
HEVs as vascular gatekeepers of TLS competence. HEV-competent niches support fresh lymphocyte influx, TLS maturation, and checkpoint-responsive immune amplification. Hypoxia, VEGF-driven angiogenesis, lactate/acidosis, and adenosine stress can impair endothelial specialization and favor poorly replenished or developmentally stalled TLS states. Direct evidence linking individual metabolic pressures to HEV differentiation within TLSs remains limited.

## Metabolic ecology of the tumor microenvironment as a determinant of TLS fate

The tumor microenvironment is metabolically abnormal, with high glycolytic flux, poor perfusion, oxygen limitation, amino-acid depletion, nucleotide breakdown, accumulation of immunoregulatory catabolites, and extracellular acidification (27, 31, 100, 101). These abnormalities do not merely impair immune cells in a diffuse manner. They create localized biochemical territories that influence stromal behavior, vascular specialization, cell-state transitions, nutrient access, and intercellular communication (27, 100–102). TLS fate must therefore be understood in relation to the metabolic neighborhood in which the structure resides (5, 29). In this review, a metabolically permissive niche does not denote the absence of metabolic activity or a single universal “normal” metabolic flux. Rather, it refers to a local environment in which perfusion and oxygenation, nutrient availability, metabolite clearance, and cell-intrinsic mitochondrial and redox fitness remain sufficient to support coordinated endothelial, stromal, dendritic-cell, T-cell, and B-cell functions, without dominant suppression by hypoxia, acidosis, extracellular adenosine, kynurenine, or lipid stress. For HEVs specifically, metabolic permissiveness denotes a vascular context compatible with endothelial specialization and sustained lymphocyte recruitment. However, the precise HEV-specific metabolic flux requirements remain incompletely defined. A central proposition of this review is that tumor-derived metabolites can shape TLS outcome by acting upstream of terminal effector failure. Instead of only weakening already activated immune cells, metabolites may interfere with dendritic-cell activation, chemokine organization, lymphocyte trafficking, Tfh–B-cell cooperation, plasma-cell differentiation, and maintenance of checkpoint-responsive T-cell states (5, 7, 12, 29, 48, 63). When several of these modules are weakened together, the result may be a histologically visible but functionally compromised TLS (5, 24, 29, 48). TLS competence is determined not only by the accumulation of suppressive metabolites but also by whether the local niche retains sufficient oxygenation, nutrient availability, metabolite clearance, and cell-intrinsic metabolic fitness to support coordinated immune, stromal, and vascular functions. **Figure 5** therefore presents metabolic permissiveness and metabolic constraint as opposing determinants of TLS fate. Current evidence points most strongly to four interconnected metabolic axes: lactate and acidosis, extracellular adenosine, hypoxia-associated vascular dysfunction, and tryptophan catabolism (24, 27, 31, 100, 101, 103–107) (**Figure 5**). Of these, tryptophan metabolism currently provides the clearest direct TLS-specific evidence. Spatial transcriptomic analysis in hepatocellular carcinoma showed that tumor-derived tryptophan metabolism restricts TLS maturation, induces deviating immature TLS states, and limits antitumor immunity, whereas inhibition of tryptophan metabolism promotes TLS maturation and synergizes with anti-PD-1 therapy (24). For lactate, adenosine, and hypoxia, mechanistic support is already strong in tumor immunology, but the TLS-specific interpretation still relies more heavily on convergent biological inference than on equally direct perturbation studies (27, 31, 100, 101, 103–106). Nevertheless, newer evidence now directly supports adenosine-mediated suppression of B-cell maturation into antibody-secreting cells, reduced plasma-cell/ASC representation in adenosine-rich tumor regions, and increased ASCs in TLS areas after A2AR blockade, thereby linking adenosine stress to impaired TLS-associated humoral output (107).

**Figure 5:**
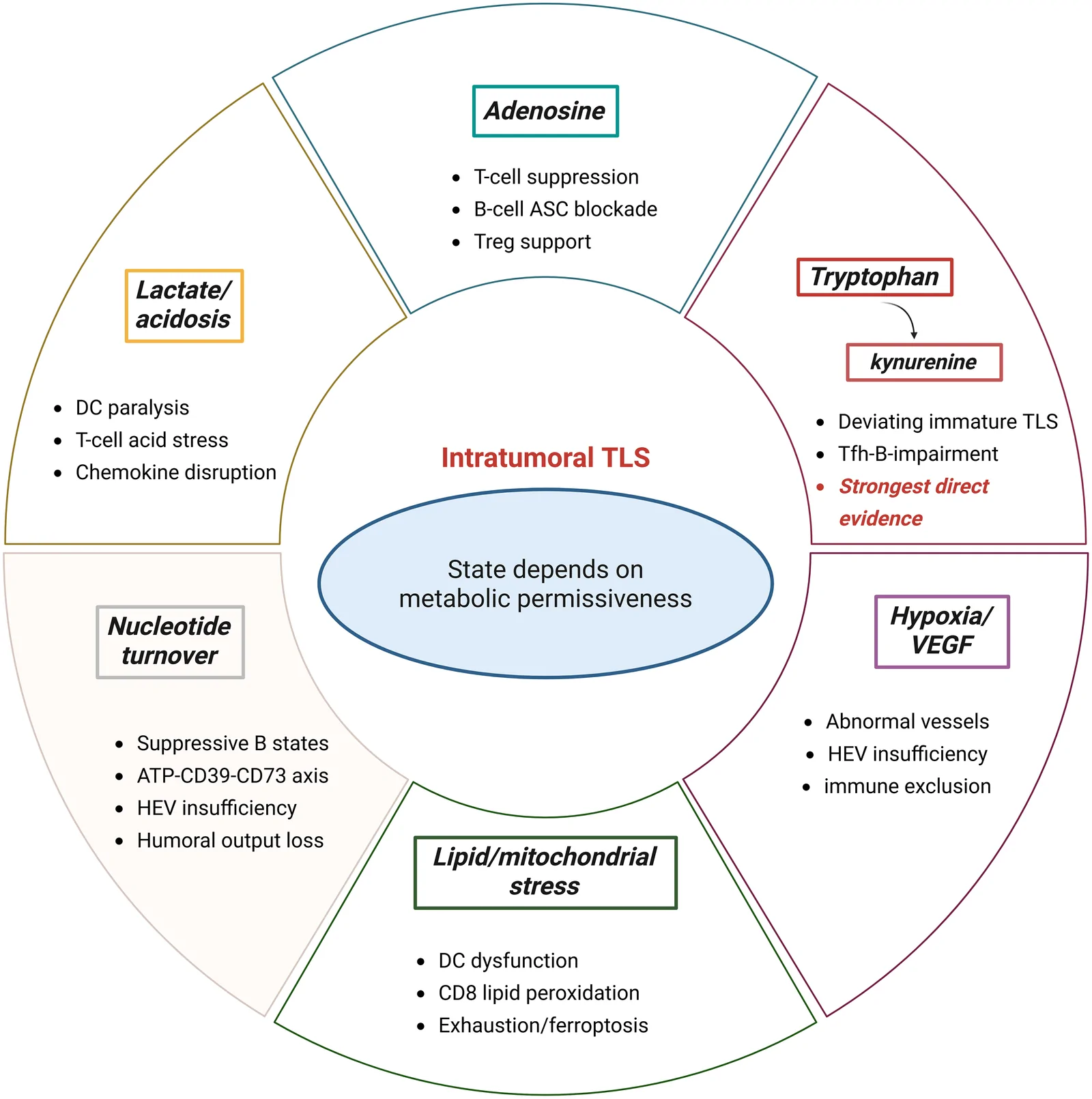
Metabolic pressures that erode TLS permissiveness and disable immune output. Metabolic permissiveness denotes the overall metabolic conditions required for coordinated HEV, dendritic-cell, Tfh–B-cell, plasma-cell, and T-cell functions. The surrounding axes represent metabolically constraining pressures that may disrupt this permissive state rather than components of metabolic permissiveness itself. Direct TLS-specific evidence is currently strongest for tryptophan metabolism, whereas several other pathways require further TLS-focused perturbation studies.

Importantly, these metabolic pathways are not supported by equivalent levels of mechanistic evidence and should not be interpreted as interchangeable drivers of TLS dysfunction. At the highest level of certainty, malignant-cell-driven tryptophan metabolism is supported by direct TLS-specific spatial mapping together with pharmacological perturbation showing altered TLS maturation and improved anti-PD-1 efficacy. Adenosine–A2AR signaling occupies an intermediate position: functional studies demonstrate suppression of T-cell responses and B-cell differentiation into antibody-secreting cells, including changes within TLS-associated regions, although complete causal effects on the whole TLS ecosystem remain incompletely established. By contrast, lactate/acidosis, hypoxia-associated vascular stress, lipid and mitochondrial dysfunction, nucleotide turnover, and nutrient competition are supported mainly by convergent evidence from individual TLS-relevant immune, stromal, or vascular modules. Their biological plausibility is strong, but direct spatially resolved perturbation studies demonstrating altered TLS maturation or output remain limited. The following sections are organized according to this hierarchy, moving from broadly supported module-level mechanisms toward pathways with stronger direct TLS-specific evidence.

### Lactate as an infrastructural suppressor of TLS competence

Lactate is a hallmark metabolite of highly glycolytic tumors and a major regulator of immune dysfunction (29, 108–110). High extracellular lactate and lactic acidosis impair dendritic-cell fitness, promote suppressive macrophage programs, and reinforce dysfunctional T-cell states (109–111). In the context of TLSs, lactate is best viewed as an infrastructural suppressor because it undermines the antigen-presenting and coordination machinery required for productive local immunity. Dendritic cells appear especially vulnerable. In melanoma, inhibition of the MCT1 lactate transporter can reprogram dendritic-cell metabolism and improve antitumor immunity (112). Because mature dendritic cells are central to antigen presentation within and around functional TLSs, these findings provide a plausible mechanistic link between lactate accumulation and TLS insufficiency (42, 112). Lactate also affects exhausted T cells through transporter-dependent uptake, increasing their sensitivity to acidic stress and limiting effective antitumor control (113). Since TLS-rich tumors often contain progenitor exhausted or stem-like T cells that seed checkpoint-responsive immunity (42, 63, 83, 114), chronic lactic stress may blunt the transition from local organization to effective effector deployment (42, 113). Direct evidence that lactate impairs B-cell differentiation specifically within TLSs remains limited, and that gap should be acknowledged (42, 110). Even so, the broader effects of lactic acidosis on cell motility, activation thresholds, cytokine programs, and antigen-presenting cell function support the idea that highly glycolytic tumors can stall TLS maturation without necessarily eliminating visible lymphoid aggregates. However, lactate-associated effects should not be regarded as uniformly suppressive or equated directly with lactic acidosis. Their biological consequences may vary according to extracellular pH, metabolite concentration, exposure duration, transporter expression, and the identity and differentiation state of the responding cell. Direct evidence that lactate preserves or improves the function of tumor-associated TLSs nevertheless remains limited.

### Extracellular adenosine: uncoupling architecture from output

The adenosine pathway is a major immunosuppressive axis in tumors. Under hypoxic, inflammatory, or tissue-damaging conditions, extracellular ATP and ADP are converted to AMP by CD39, followed by CD73-mediated generation of extracellular adenosine. Among the four adenosine receptors, the high-affinity A2A adenosine receptor (A2AR; encoded by ADORA2A) is particularly important in regulating antitumor T-cell responses. A2AR engagement activates Gs–adenylyl cyclase–cAMP–PKA signaling, thereby increasing the activation threshold of T cells and suppressing T-cell receptor-dependent proliferation, cytokine production, and cytotoxic effector activity. Experimental deletion or pharmacological blockade of A2AR enhances antitumor T-cell function, while combined A2AR and PD-1 blockade increases IFN-γ and granzyme B expression in tumor-infiltrating CD8-positive T cells and improves tumor control. Adenosine–A2AR signaling can also impair the metabolic fitness of human peripheral and tumor-infiltrating CD8-positive T cells, linking extracellular purine accumulation to both functional and metabolic T-cell suppression (115–117).

These effects are particularly relevant to TLS-bearing tumors, in which continuous recruitment and local expansion of stem-like or progenitor exhausted T cells contribute to checkpoint-responsive immunity. An adenosine-rich niche could therefore restrict the proliferation and effector differentiation of newly recruited T cells and weaken the helper signals required for productive Tfh–B-cell cooperation. However, direct spatial evidence demonstrating A2AR-dependent suppression of T cells specifically within intratumoral TLSs remains limited, and this connection should currently be interpreted as a mechanistically supported TLS-focused hypothesis rather than established TLS-specific causality. Beyond T cells, recent evidence indicates that A2AR signaling directly inhibits B-cell maturation toward antibody-secreting cells through a B-cell-intrinsic mechanism, whereas A2AR blockade restores immunoglobulin production and increases antibody-secreting-cell representation in TLS-associated regions (116, 117). Thus, extracellular adenosine may not necessarily eliminate TLS architecture but may uncouple visible lymphoid organization from both effective T-cell amplification and plasma-cell output.

### Tryptophan catabolism: the clearest evidence for metabolite-driven TLS deviation

Among currently implicated metabolic pathways, tryptophan catabolism provides the strongest direct evidence that metabolites can alter TLS developmental fate. In hepatocellular carcinoma, near-single-cell spatial transcriptomics identified distinct immature TLS subtypes termed conforming and deviating (24). Conforming TLSs retained niche functions resembling mature TLSs and were associated with immunotherapy-responsive states, whereas deviating TLSs were not. Critically, malignant cell-driven tryptophan metabolism was linked to this deviation, and inhibition of tryptophan metabolism promoted TLS maturation, improved tumor control, and synergized with anti-PD-1 therapy (24). This study is pivotal because it shows that the metabolic niche can shape TLS trajectory rather than merely suppress immunity after maturation. It also provides pharmacological evidence that metabolically constrained TLSs can be redirected toward a more functional state. Earlier work on IDO1 and kynurenine biology further strengthens this interpretation. In inflamed non-small cell lung cancer, kynurenine impaired Tfh-induced differentiation of antibody-producing B cells, directly implicating tryptophan catabolism in the humoral engine of TLSs (118). Taken together, these findings make tryptophan metabolism the clearest current example of metabolite-driven TLS failure.

### Hypoxia, aberrant angiogenesis and vascular stress

Hypoxia is a dominant organizing condition in solid tumors and lies upstream of several other suppressive pathways, including lactate accumulation, extracellular acidosis, VEGF-driven angiogenesis, and adenosine generation (108, 119–121). It profoundly affects stromal remodeling, vascular patterning, endothelial activation, and immune trafficking (105, 106, 108). Functional TLSs require HEVs and other inflammatory vascular programs that support lymphocyte recruitment, replenishment, and spatial organization, whereas hypoxia-driven angiogenesis typically produces structurally abnormal, poorly perfused, leaky, and immunologically dysfunctional vessels (49, 51, 98, 105, 106). Although direct TLS-focused hypoxia-intervention studies remain limited, convergent evidence from vascular biology and tumor immunometabolism supports a model in which hypoxia erodes the vascular and stromal prerequisites of TLS competence (49, 50, 76, 105, 106, 108, 122). A tumor may therefore induce partial lymphoid aggregation without establishing the endothelial gateway needed for efficient recruitment, replenishment, and local organization (49, 51, 98). In this framework, hypoxia may not prevent lymphoid aggregation altogether, but may favor the emergence of incompletely vascularized, poorly replenished, or developmentally stalled TLS states.

### Emerging metabolic and cellular layers: lipid stress, nucleotide metabolism, and suppressive B-cell states

Beyond the four best-characterized axes, several emerging layers deserve explicit attention. Lipid uptake and mitochondrial rewiring can shape macrophage, dendritic-cell, and T-cell states, thereby altering the support network on which TLSs depend (31, 123–126). These pathways are unlikely to act as isolated “TLS switches”; rather, they may condition antigen-presenting cells, stromal organizers, lymphocyte persistence, and survival thresholds in spatially restricted niches (5, 31, 48, 123). In cancer, lipid accumulation in dendritic cells can impair antigen-processing and T-cell-stimulatory capacity, while CD36-dependent uptake of fatty acids or oxidized lipids in CD8^+^ T cells promotes lipid peroxidation, ferroptosis, and functional exhaustion, providing plausible routes through which lipid-rich niches could weaken TLS competence (124–126). Nucleotide metabolism is similarly relevant because extracellular ATP release, CD39/CD73 activity, and adenosine accumulation connect tissue damage, hypoxia, vascular dysfunction, and humoral immune output (104, 107, 127). Future spatial studies should therefore integrate metabolic enzyme expression, metabolite imaging, vascular markers, and lymphocyte-state annotation in the same tissue sections, especially because spatial transcriptomic studies already show that metabolic neighborhoods can distinguish productive from developmentally deviated TLS states (5, 24, 29).

A second emerging layer is cellular rather than purely biochemical. Recent work shows that selected B-cell states can disrupt TLS integrity and impair immunotherapy responses by targeting HEV-associated lymphotoxin signaling, indicating that B-lineage cells are not uniformly protective in TLS-positive tumors (72). Specifically, IGLL5^+^ B cells in bladder cancer were shown to bind HEVs through IGLL5-LTβR ligand-receptor interactions, inhibit non-canonical NF-κB signaling, promote TLS disassembly, and reduce immunotherapy efficacy (72). This observation strengthens the central argument of this review: TLS competence is a state-dependent ecological property. A tumor can contain B cells, HEVs, and lymphoid aggregates while still losing the regulatory circuitry required for productive local immunity (5, 12, 13, 48, 72). For clinical translation, this means that therapies should aim to preserve or restore the right B-cell, germinal-center, and plasma-cell programs, not simply increase total B-cell infiltration (7, 12, 13, 48).

## Metabolite-driven TLS failure as a mechanism of immune escape

Cancer immune escape is commonly explained through antigen loss, defective antigen presentation, checkpoint signaling, myeloid suppression, regulatory-cell expansion, and terminal T-cell exhaustion. These mechanisms remain fundamental. However, the emerging TLS literature adds another dimension: tumors may also evade immune control by preventing the formation, maturation, or persistence of effective local immune-organizing centers (41, 42, 65). This framework helps explain why some tumors rich in lymphoid aggregates still resist checkpoint blockade. The decisive variable may not be TLS quantity, but whether the structures are developmentally advanced, metabolically permissive, and coupled to appropriate T-cell and B-cell states (24, 41, 42, 65, 84). At the same time, TLS competence should not be viewed as universally sufficient for therapeutic success, because even tumors containing mature TLSs may remain resistant when the surrounding niche is dominated by suppressive stromal programs, immune exclusion, or deeply dysfunctional T-cell states (64). This helps explain why mature TLS morphology alone cannot fully predict response to checkpoint blockade. In renal cell carcinoma, response to PD-1 blockade depends not only on TLS abundance, but also on a favorable surrounding T-cell state (84). In hepatocellular carcinoma, metabolic pressure can divert immature TLSs away from productive maturation (24). Under this view, metabolite-driven TLS dysfunction acts as a higher-order mechanism of immune escape that limits the local context in which antigen presentation, T-cell reactivation, B-cell differentiation, and clonal propagation would otherwise occur. Checkpoint blockade can release inhibitory signaling on pre-existing T cells, but it may not fully compensate for a microenvironment that has already prevented the establishment of a competent intratumoral immune organ (24, 65, 84). The relevant question is therefore whether a tumor supports a functional TLS ecosystem or only a stalled lymphoid remnant.

## Translational implications: biomarkers and therapeutic design

A major translational implication of this framework is that TLSs should be evaluated as multidimensional indicators of immune-niche competence rather than as binary histological features (4, 5, 16, 18). A submission-ready biomarker framework should include at least six layers: spatial location, structural maturity, HEV status, immune-cell state composition, B-cell/plasma-cell output, and metabolic context (5, 18, 57, 90). Spatial location distinguishes deeply intratumoral TLSs from peritumoral or stromal-border aggregates (128, 129). Structural maturity captures T/B-cell segregation, FDC organization, germinal-center-like activity, and HEV support (5, 18, 54). Cellular composition should report activated B cells, plasmablasts/plasma cells, mature dendritic cells, Tfh-like cells, progenitor-like or stem-like T cells, suppressive B-lineage states, and terminally dysfunctional T-cell states (7, 20, 74, 90, 130). Metabolic context should include at least lactate transport/acidosis, adenosine signaling, tryptophan catabolism, hypoxia-associated vascular stress, and lipid or mitochondrial programs where data are available (24, 49, 131–134). These dimensions are more informative than TLS counts alone because they ask whether a structure is likely to function as a productive immune niche or merely persist as a recognizable but ineffective remnant (**Table 2**; **Figure 6**).

**Table 2:** Proposed multidimensional TLS competence biomarker framework for immunotherapy-oriented studies.

Biomarker layer	What to score	Competent TLS features	Dysfunctional/metabolic bottleneck	Suggested assays
Spatial location	Intratumoral, peritumoral, stromal-border, TLS-desert pattern	TLSs embedded in tumor bed and linked to immune-inflamed neighborhoods	Spatial exclusion, border-restricted aggregates, necrotic/hypoxic adjacency	H&E/IHC, multiplex IF/IHC, spatial transcriptomics
Structural maturity	T/B segregation, FDC network, GC-like activity, follicular architecture	FDC^+^, GC-like B-cell zones, organized T-cell and B-cell compartments	Loose aggregates, absent FDCs, no GC-like reaction	CD20/CD3, CD21/CD23, BCL6, AID, Ki-67, digital pathology
HEV status	HEV density, maturity, and proximity to TLS and tumor nests	PNAd+/MECA-79^+^, CCL21^+^, lymphocyte-entry phenotype	HEV-poor, hypoxic or VEGF-dominated abnormal vessels	PNAd/MECA-79, CCL21, endothelial panels, vascular spatial maps
Immune-cell state composition	mDCs, Tfh-like cells, progenitor exhausted T cells, Tregs, terminally exhausted cells	mature DC support, Tfh-like help, TCF1^+^ or progenitor-like T-cell niches	tolerogenic DCs, excess Tregs, terminal dysfunction, suppressive myeloid states	scRNA-seq/snRNA-seq, multiplex imaging, TCR analysis
B-cell/plasma-cell output	Naive/memory/GC-like B cells, plasmablasts, plasma cells, immunoglobulin programs	class switching, local clonal expansion, IgG^+^ plasma-cell output	extrafollicular or suppressive B programs, ASC blockade, poor Ig output	BCR-seq, Ig transcript/protein assays, CD20/CD38/CD138 panels
Metabolic context	Perfusion and oxygenation; nutrient availability; lactate/acidosis; extracellular adenosine; tryptophan/kynurenine metabolism; hypoxia/VEGF-associated vascular stress; lipid, mitochondrial, and redox programs	Adequate perfusion and oxygenation, balanced nutrient availability, effective metabolite clearance, limited accumulation of suppressive metabolites, and preserved cell-intrinsic mitochondrial and redox fitness supporting HEV, dendritic-cell, and Tfh–B-cell modules	lactate-rich, adenosine-rich, kynurenine-high, hypoxic/VEGF-high, lipid-stressed niches	Spatial transcriptomics; targeted or spatial metabolomics; hypoxia, pH, and vascular markers; and metabolic enzyme, transporter, receptor, mitochondrial, and redox panels
Therapeutic match	Dominant bottleneck and reversibility	bottleneck-specific rescue of TLS function	indiscriminate TLS induction without restoring output	biomarker-guided combination trials with pre/on-treatment biopsies

**Figure 6:**
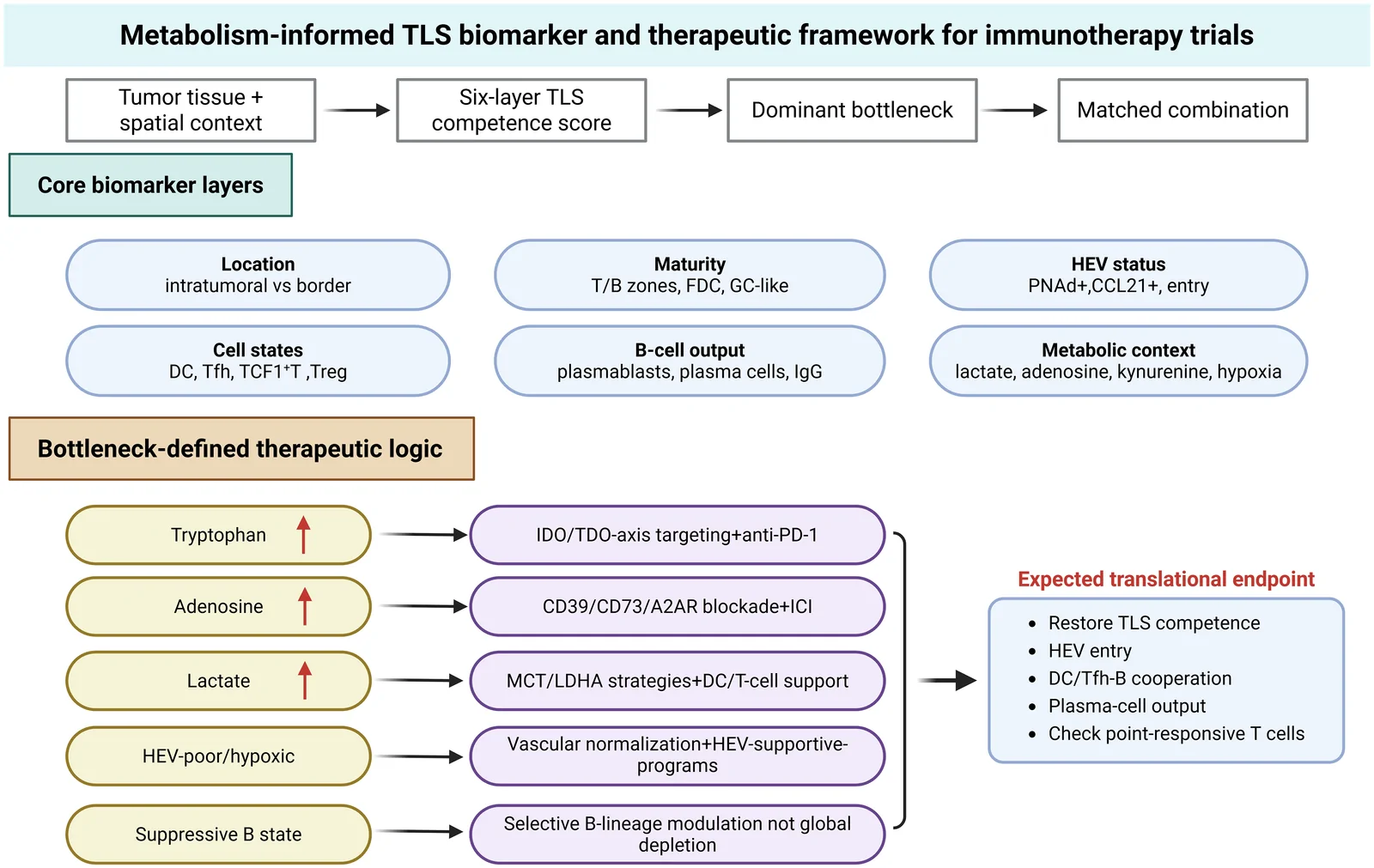
Translational framework for metabolism-informed TLS scoring and therapy. Spatially resolved TLS scoring should define the dominant bottleneck and guide rational combinations, such as tryptophan-axis inhibition, adenosine-pathway blockade, lactate-transport or LDHA-targeting strategies, vascular normalization, or selective B-lineage modulation together with immunotherapy.

A practical next step will be to translate this ecological framework into reproducible tissue-based scoring systems (18, 57). Beyond simple TLS counting, future pathology-oriented approaches should incorporate several core dimensions: TLS relative area or abundance, intralesional B-cell density, evidence of FDC organization, HEV presence, plasma-cell output, and spatial localization relative to tumor nests and stromal interfaces (7, 18, 49, 57, 130). Such multidimensional scoring may provide a more functional approximation of TLS competence and may improve patient stratification for immunotherapy (11–13, 16, 18, 57). Importantly, however, biomarker development will require standardization across platforms and tumor types (18, 57). The field still lacks consensus thresholds for defining maturity, dysfunction, or metabolic restriction, and without such harmonization there is a risk of converting a biologically rich concept into another overly simplified pathology metric.

Spatial omics offers a particularly powerful route for operationalizing this next-generation TLS pathology. By combining spatial transcriptomics, multiplex imaging, and immune receptor repertoire analysis, it should become possible to map TLS architecture, cellular interaction networks, clonal dynamics, and local metabolic programs within the same tissue context (20, 24, 135). This could help identify composite biomarker signatures that distinguish productive TLS ecosystems from developmentally stalled or metabolically constrained states (20, 24, 74). At the same time, practical deployment will require careful attention to assay scalability, analytic reproducibility, and pathology integration; otherwise, the translational promise of spatially resolved TLS profiling may remain largely academic (18, 20, 57, 135).

Therapeutically, the most practical implication is not to “induce TLSs” indiscriminately, but to rescue the missing module that prevents a TLS from becoming competent (5, 24, 49). In HCC, inhibition of tryptophan metabolism restored TLS maturation and improved anti-PD-1 efficacy, providing a clear proof of principle for metabolic redirection (24). In other settings, the dominant bottleneck may differ: lactate-rich tumors may require dendritic-cell reprogramming or lactate-transport blockade (131, 133, 134); adenosine-rich tumors may require CD39/CD73/A2AR targeting (132); hypoxic tumors may require vascular normalization or HEV-supportive stromal reprogramming (49); and tumors enriched in suppressive B-lineage states may require selective modulation rather than global B-cell depletion (74, 90). These strategies should be developed with matched tissue biomarkers (18, 24, 57). Without patient selection, TLS-directed metabolic therapy risks either missing the relevant bottleneck or increasing nonspecific inflammation without restoring functional antigen presentation, lymphocyte recruitment, and plasma-cell output (7, 24, 49).

## Outstanding questions and future directions

Several questions define the next phase of TLS research. First, the strength of causal evidence remains uneven across candidate metabolic pathways. Among these, tryptophan catabolism currently has the strongest direct support for a role in TLS deviation (24), whereas the cases for lactate, adenosine, and hypoxia depend more on mechanistic plausibility and convergent inference from broader tumor immunology (131–134). This does not weaken the hypothesis, but it does require a more disciplined framing of certainty. The field now needs perturbation studies that directly test how specific metabolites alter TLS initiation, maturation, maintenance, and output across tumor models and human tissues.

A second challenge is conceptual and methodological standardization. The terminology used to describe dysfunctional TLS states remains inconsistent, with labels such as immature, deviating, dysfunctional, non-productive, and stalled often used interchangeably despite potentially distinct biology (4, 5, 18, 24, 90). A useful framework should distinguish at least three levels: structural maturity, functional competence, and ecological permissiveness (4, 5, 16, 18, 57, 90). Without such separation, descriptive pathology, mechanistic biology, and translational biomarker work will continue to talk past one another. Equally important, future studies will need standardized spatial and molecular criteria for annotating HEVs, FDC networks, germinal center activity, plasma-cell output, and metabolic restriction (7, 18, 20, 49, 57, 130, 135).

An additional unresolved question is whether TLS failure in advanced disease is fully reversible. Current evidence suggests that biological rescue is possible, but its success is likely to depend on timing, tumor type, vascular integrity, and the degree of immune exhaustion already present (16, 24, 49). A TLS that is absent, a TLS that is structurally immature, and a TLS that is morphologically mature but functionally suppressed may not respond to the same intervention (16, 18, 24, 90). This implies that future therapies may need to be tailored not only to tumor genotype or immune phenotype, but also to TLS state (16, 18, 57, 90). In parallel, longitudinal studies will be needed to determine whether metabolically constrained TLSs can be durably reprogrammed or whether they repeatedly regress under persistent tumor pressure (20, 24, 135).

Several limitations should be kept in mind when interpreting the current literature. Much of the available evidence remains correlative, and spatial co-localization between metabolic programs and dysfunctional TLS states does not, by itself, establish causality (4, 5, 24). Moreover, metabolic pathway scores derived from single-cell or spatial transcriptomic data reflect inferred metabolic potential rather than direct measurements of metabolite abundance, enzyme activity, substrate utilization, or pathway flux. Direct metabolite measurements and, where feasible, isotope-tracing or functional flux analyses are therefore required to validate metabolism-associated TLS classifications. Many studies also infer TLS functionality from surrogate markers rather than direct measurements of antigen presentation, clonal evolution, antibody production, or therapeutic responsiveness (7, 20, 130, 135). In addition, tumor type, sampling strategy, treatment exposure, and analytic platform all influence how TLSs are detected and interpreted (5, 18, 57, 63, 136–138). These limitations do not diminish the importance of the emerging framework, but they argue against overgeneralization and underscore the need for more rigorous cross-platform and cross-tumor validation. Platform-specific biases in dissociation, spatial resolution, tissue fixation, cell segmentation, and deconvolution should be reported because they can change whether a TLS is classified as mature, deviating, or metabolically constrained.

## Conclusions

TLS research has moved from asking whether TLSs matter to asking why some TLSs sustain antitumor immunity whereas others stall. Single-cell and spatial omics now show that TLSs are dynamic ecological units whose competence depends on stromal, vascular, myeloid, T-cell, and B-cell programs embedded within a metabolically permissive niche. Metabolite-driven TLS dysfunction therefore represents a plausible and increasingly testable mechanism of cancer immune escape. The current evidence is strongest for tryptophan catabolism, while lactate, adenosine, hypoxia, lipid remodeling, and suppressive B-lineage states require more direct TLS-focused perturbation studies. The next step is to stop counting TLSs in isolation and instead define their state, bottleneck, and reversibility. In clinical biopsies, this could begin with a scalable multiplex panel integrating T/B-cell organization, HEV and germinal-center-like maturation, plasma-cell output, and a biomarker matched to the dominant metabolic bottleneck. A metabolism-informed TLS framework may improve patient stratification and guide therapies that restore intratumoral immune competence rather than merely document its failure.
